# DNA methylation affects metastasis of renal cancer and is associated with TGF-β/RUNX3 inhibition

**DOI:** 10.1186/s12935-018-0554-7

**Published:** 2018-04-10

**Authors:** Jianbo Zheng, Yanhui Mei, Ping Xiang, Guangsheng Zhai, Ning Zhao, Chuanbing Xu, Min Liu, Zhengsheng Pan, Kai Tang, Dongsheng Jia

**Affiliations:** 1 Department of Urology, QiLu Hospital of Shandong University, 107 Wenhua Western Road, Jinan, 250012 Shandong Province China; 2 Department of Urology, Central Hospital of Zibo, No. 54 Gongqingtuan West Road, Zhangdian District, Zibo, 255036 Shandong Province China; 3 Department of Urology, Binzhou Medical University Hospital, No 2 Huanghe Road, Binzhou, 256603 Shandong Province China; 4 Department of Urology, Anhui Provincial Hospital, 17 Lujiang Road, Hefei, 230001 Anhui Province China; 5 Department of Radiotherapy, Central Hospital of Zibo, No. 54 Gongqingtuan West Road, Zhangdian District, Zibo, 255036 Shandong Province China

**Keywords:** Renal cancer, Methylation, RUNX3, TGF-β, Metastasis, CpG, Xenograft model

## Abstract

**Background:**

Renal cell carcinoma accounts for 2–3% of all cancers and metastasis increased the malignancy of renal cancer. However, the role of methylation in metastasis of renal cancer is poorly understood.

**Methods:**

We performed targeted gene array to compare the differential expressions of methylation regulated genes in metastatic and primary renal cancer tissues. Quantitative methylation specific PCR was performed to examine the CpG methylation levels of Runt related transcription factor 3 (RUNX3) and transforming growth factor (TGF)-β. Western blot was performed to detect the expression of target genes. Murine xenograft renal cancer model was established to assay gene expression, methylation level, tumor growth and animal survival in vivo.

**Results:**

RUNX3 and TGF-β levels were decreased in metastatic renal cancer tissues as a result of their CpG methylation. Metastatic xenograft model displayed decreased expression levels of RUNX3 and TGF-β and higher CpG methylation levels, bigger tumor size and shorter survival time, all which were restored by treatment with a methylation inhibitor.

**Conclusions:**

Hypermethylation in CpG islands promotes metastasis of renal cancer and is associated with TGF-β and RUNX3 inhibition.

**Electronic supplementary material:**

The online version of this article (10.1186/s12935-018-0554-7) contains supplementary material, which is available to authorized users.

## Background

Renal cancer, or renal cell carcinoma, accounts for 2–3% of all cancers [1]. As a result of advances in assisted screening and growing concern about health, the incidence rate of incidental, newly diagnosed renal cancer has risen by 60%, while nearly 50% of new cases are found in routine physical examination or ultrasound for other diseases. However, about 25% of new cases metastasize at diagnosis [2]. The occurrence and development of renal cancer are complex processes of polygenic changes, and two main categories of mechanisms are involved: (1) genetic mechanism, where the DNA nucleotide sequence changes to form mutations causing tumor formation; (2) epigenetics mechanism, where the DNA nucleotide sequence remains unchanged but nucleotide modifications cause changes in gene expression levels. For example, abnormal DNA methylation influences the transcription of genes related to renal cancer, thus affecting the genesis of renal cell carcinoma [3].

Runt-related transcription factor 3 (RUNX3) is a novel tumor suppressor gene discovered in recent years. There are reports demonstrating that RUNX3 plays a role in cancer development. For instance, the high expression of RUNX3 could inhibit tumor microvascular generation in order to exhibit negative control response on invasion and distant metastasis in colorectal adenocarcinoma [4]. It was reported recently that abrogation of RUNX3 expression inhibited bone invasion in oral squamous cell carcinoma. RUNX3 may be a useful predictive biomarker and therapeutic target for bone invasion of oral cancer [5].

Studies have shown that methylation of RUNX3 is a risk factor for the development of lung cancer and subsequent tumor progression [6]. In addition to lung cancer, there are more reports suggesting that RUNX3 can inhibit tumor development by affecting the growth, metastasis, and infiltration of renal cancer cells [7–9]. However, the relationship between RUNX3 and methylation in renal cancer has not been studied.

In addition, it has been reported that RUNX3 may be a link in the transforming growth factor (TGF)-β pathway [10, 11]. TGF-β superfamily proteins have many important biological functions, including regulation of tissue differentiation, cell proliferation and migration in both normal and cancer cells [12]. It is well known that TGF-β is involved in both suppressive and inflammatory immune responses [13]. Importantly, TGF-β is a transforming growth factor that inhibits growth and induces apoptosis. The signaling disorder of TGF-β leads to the development of many tumors [14]. For example, TGF-β1 was reported to regulate the pattern of hMENA to modulate cell invasion in pancreatic cancer [15]. Moreover, TGF-β is associated with methylation [16].

Immunotherapy is standard care for patients with renal cancer, and chemotherapy has also been shown to be useful. However, occasional low sensitivity for radiotherapy and chemotherapy is a problem. In this study, we proposed that methylation inhibited TGF-β**/**RUNX3 pathway to affect the metastasis in renal cancer. The aim of this study was to investigate the relationship between methylation and renal cancer, as well as the pathways affected by methylation, in order to apply the methylation regulation of tumor suppressor genes as an adjuvant therapy for renal cancer. We investigated the methylation levels and expression differences of RUNX3 and TGF-β in metastatic renal cancer and primary renal cancer, as well as the roles of their methylation in a metastatic renal cancer xenograft model.

## Materials and methods

### Murine xenograft renal cancer model

Experiments with animals involved were conducted following the guidelines for animals approved by the Committee on the Ethics of Animal Experiments of QiLu Hospital of Shandong University and Central Hospital of Zibo. The metastatic or primary renal cancer cells (1 × 10^4^) were suspended in 50 µL of culture medium and injected into the left foreleg of the BALB/c nude male mice (purchased from Charles River, Beijing, China) aged at 6–8 weeks. As indicated, some metastatic renal cancer cells were treated with 5-aza-2′-deoxycytidine (10 mM, Sigma, St. Louis, MO, USA) for 2 days and then were injected to the left foreleg of the BALB/c nude male mice with the untreated cells as the control. The animals were monitored daily until death, and the survival days after inoculation were calculated. Animals used for tumor size analysis were euthanized at 2 months post inoculation, and tumors were dissected with size determined using a caliper. Then the tumor tissues were used for methylation and gene expression analysis.

### Renal cancer patient samples

All experiments complied with the regulations and guidelines and were approved by the ethics committee of QiLu Hospital of Shandong University and Central Hospital of Zibo. All renal cancer patients involved in this study provided written informed consent to participate in this study (Table 1 and Additional file 1: Table S1). The patients underwent the surgery to remove the renal cancer tissue, which were subjected to extraction of DNA and proteins for methylation analysis and Western blot.

**Table 1:** Patient characteristics (primary n = 53; metastasis n = 67)

Characteristics	Primary	Metastasis	p value
Age (years)			0.876
< 60	19	20	
> 60	48	33	
Gender			0.902
Female	31	32	
Male	28	29	
Tumor size (cm)			0.000*
< 5	27	41	
> 5	34	17	
Tumor metastasis			0.000*
Negative	53	0	
Positive	0	67	
CpG methylation level			0.000*
Low expression	24	3	
High expression	29	64	
Global methylation level			0.000*
Low expression	30	59	
High expression	23	8	

Statistical analyses were performed by the SPSS test. *p < 0.01 was considered significant

### Cell culture

Primary renal patient cancer tissues and metastatic disease tissues (from the bone, lymph, liver and lung of patients) were cut to small pieces by spring scissors, followed by collagenase treatment. After that, the mixture was filtered by 100 and 40 µM cell strainer sequentially, and centrifuged at 400*g* for 10 min. Finally, the cell pellet was suspended in Dulbecco’s Modified Eagle Medium (DMEM) culture media supplemented with 10% fetal bovine serum (FBS) and Pen/Strep (GIBCO-Invitrogen, 1:100). Renal cancer cells were maintained in DMEM culture media with 10% FBS at 37  °C in a humidified incubator with 5% CO_2_.

### Gene arrays

Targeted quantitative RT-PCR arrays were performed to measure expression of genes with CpG islands in their promoters. The objective was to investigate the methylation regulated genes associated with metastasis, rather than the entire database of human genes.

### Quantitative methylation specific PCR (qMSP)

Quantitative methylation specific PCR was performed as previously described [17]. In brief, purified DNA samples from patient cancer tissues or xenograft tumors were bisulfite-converted with EZ-96 DNA Methylation-GoldTM Kit (Zymo Researh). 10 ng bisulfite-converted DNA was then used for pre-amplification with gene specific primers and probed with 1× Taqman Universal PCR master mix (ThermoFisher Scientific) in 15 µL reaction for 15 cycles. Next, 2 µL pre-amplification product was used for qMSP analysis in 10 µL reaction system with the same primers as in pre-amplification and 1× Taqman Universal PCR master mix for 50 cycles in a 7900HT Fast RealTime PCR System (Applied Biosystems). All qMSP reactions were run in triplicates. The Alu-element based normalization assay for qMSP was conducted as quantity and quality control. RUNX3 and TGF-β detection limit was examined by decreasing amounts of methylated control DNA mixed into unmethylated control DNA. Primers used in this study were as follows: RUNX3 sense 5′-GGC AAT GAC GAG AAC TAC-3′, antisense 5′-GGA GAA TGG GTT CAG TTC-3′; TGF-β sense 5′-CAC CCG CGT GCT AAT GG-3′, antisense 5′-ATG CTG TGT GTA CTC TGC TTG AAC-3′; β-actin sense 5′-AGA GCT ACG AGC TGC CTG AC-3′, antisense 5′-AGC ACT GTG TTG GCG TAC AG-3′.

### Western blot

Equal amounts of protein samples (20 μg) were loaded in an 8–12% SDS–polyacrylamide gel and then were transferred to a polyvinyl-iodine fluoride membrane (Biorad, Hercules, CA, USA). After blocking (5% non-fat dry milk), membranes were incubated with primary antibodies overnight at 4 °C (rabbit anti-RUNX3, Abcam ab11905, 1:3000, rabbit anti-TGF-β, R&D systems AB-100-NA, 1:1000, mouse anti-β-actin, R&D systems MAB8929, 1:1000) followed by 1 h incubation with corresponding secondary anti-mouse (1:5000, Sigma) or anti-rabbit (1:5000, Sigma) antibodies conjugated to HRP. Chemiluminescent visualization was performed by SuperSignal West Pico Chemiluminescent Substrate (Thermo Scientific, Waltham, MA, USA) with β-actin as the internal loading control.

### Statistical analyses

All data shown were repeated in at least three independent experiments and are presented as mean ± SD. Student’s *t* tests were used to analyze the differences between two groups. *P* < 0.05 was considered significant.

## Results

### Elevation of CpG methylation in metastatic renal cancer tissues

In this study, we utilized metastatic and non-metastatic/primary renal cancer tissues from patients. Metastatic renal cancer tissues were obtained from 67 patients (Additional file 1: Table S1) and primary renal cancer tissues were obtained from 53 patients (Table 1). The level of CpG methylation was elevated in patients with metastatic renal cancer, while the level of global methylation was decreased in metastatic renal cancer. It indicated that high CpG methylation was related to metastatic feature of renal cancer.

### Downregulated RUNX3 and TGF-β in metastatic renal cancer tissues

**Fig. 1:**
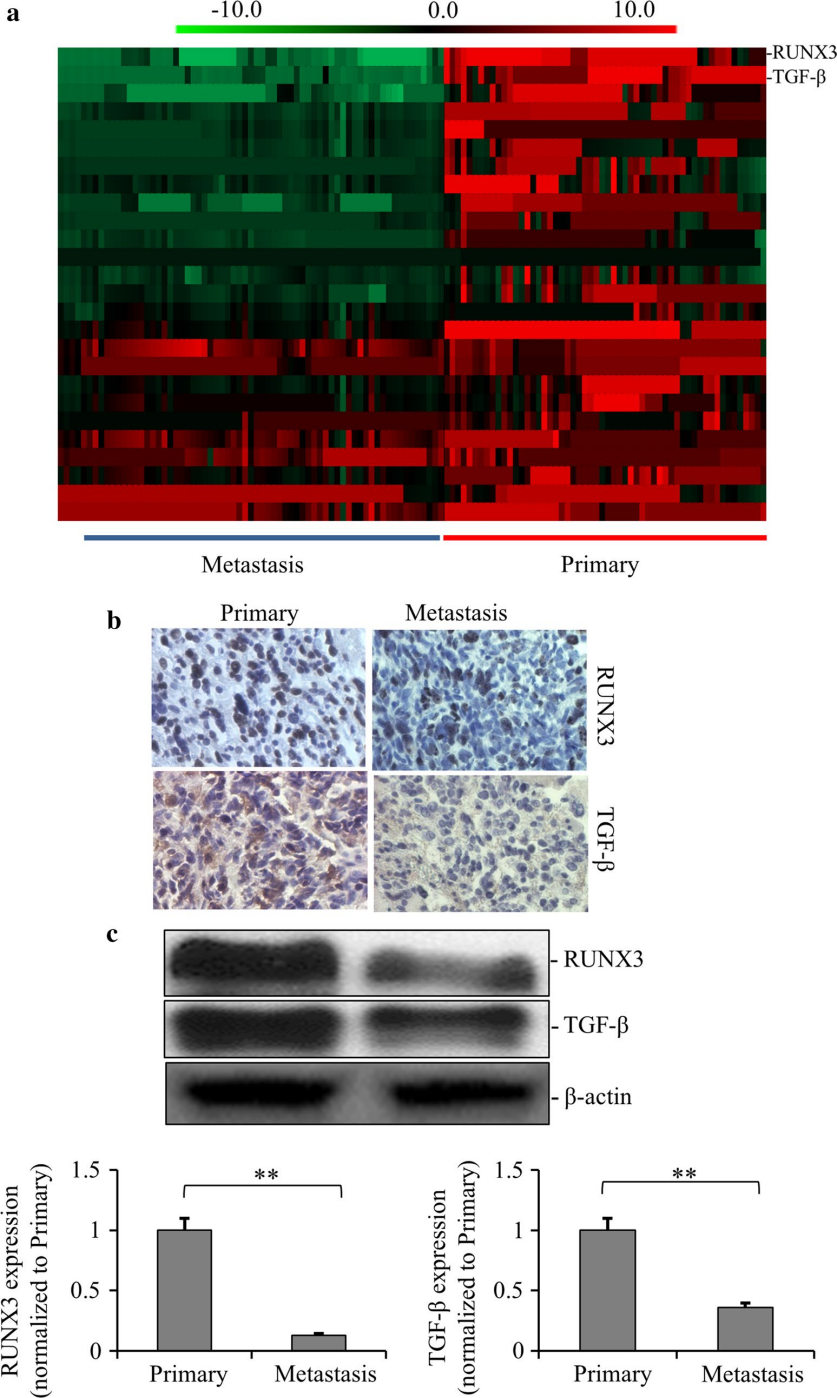
The list of changed genes in metastatic renal cancer tissues from 67 patients vs. 53 primary renal cancer tissues from the patients. **a** The heatmap of differentially expressed genes identified in metastatic renal cancer compared with primary renal cancer. The decreased and increased genes are indicated by different intensities of green and red, respectively. RUNX3 and TGF-β were the top 2 down-regulated genes. **b** The expressions of RUNX3 and TGF-β in metastatic renal cancer compared with the primary renal cancer were examined by immuno-histochemical staining with 20X magnification. **c** The expression of RUNX3 and TGF-β in metastatic renal cancer compared with the primary renal cancer was examined by Western blot. Relative expression values represent mean ± SD from three independent experiments. Quantitation by densitometry is also shown. **p < *0.01*

In order to identify the differentially expressed genes regulated by methylation between metastatic and primary renal cancer, gene array was performed. Figure 1a showed the heatmap of differentially expressed genes identified in metastatic renal cancer compared with primary renal cancer in the order of most changed genes on the top. As shown, most differentially expressed genes were downregulated, with RUNX3 and TGF-β being the top 2 down-regulated genes. Furthermore, the decreased expressions of RUNX3 and TGF-β in metastatic renal cancer compared with the primary renal cancer were confirmed by Western blot (Fig. 1b).

### Increased CpG methylation of RUNX3 and TGF-β in metastatic renal cancer tissues

**Fig. 2:**
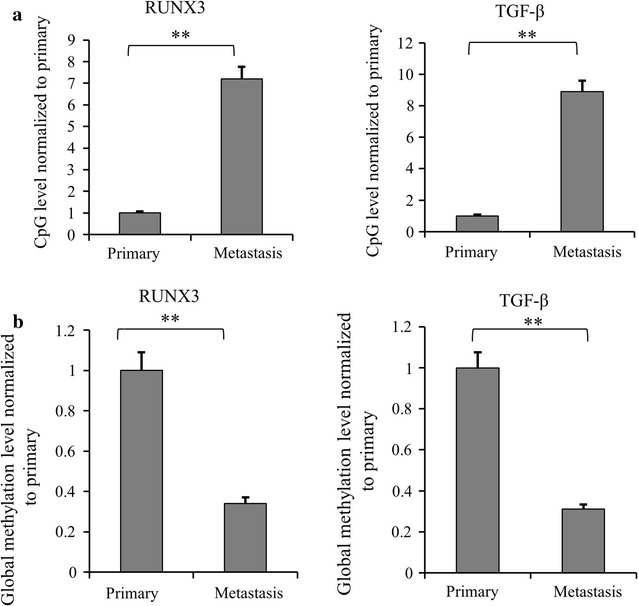
The change of methylation levels of RUNX3 and TGF-β in metastatic renal cancer vs. primary renal cancer from patients. **a** CpG methylation levels of RUNX3 and TGF-β in metastatic cancer tissues were detected by qMSP compared with primary renal cancer tissues. The data were mean ± SD from three independent experiments (**p < *0.01*). **b** Global DNA methylation levels of RUNX3 and TGF-β in metastatic renal cancer tissues were detected using quantitative methylation real-time PCR compared with primary renal cancer tissues. The data were mean ± SD from three independent experiments (**p < *0.01*)

Next, we compared the changes in methylation levels of RUNX3 and TGF-β in metastatic renal cancer tissues, with primary renal cancer tissues as the control. As shown in Fig. 2a, elevated CpG methylation levels of RUNX3 and TGF-β were detected in metastatic cancer tissues by qMSP, while reduced global DNA methylation levels of RUNX3 and TGF-β were observed compared with primary renal cancer tissues by quantitative methylation real-time PCR (Fig. 2b). It showed 7 to ninefolds of CpG methylation level, and the global methylation levels were reduced to ~ 30% in metastatic renal cancer, as compared to primary renal cancer tissues.

### Reduced expression of RUNX3 and TGF-β in metastatic renal cancer xenograft model

**Fig. 3:**
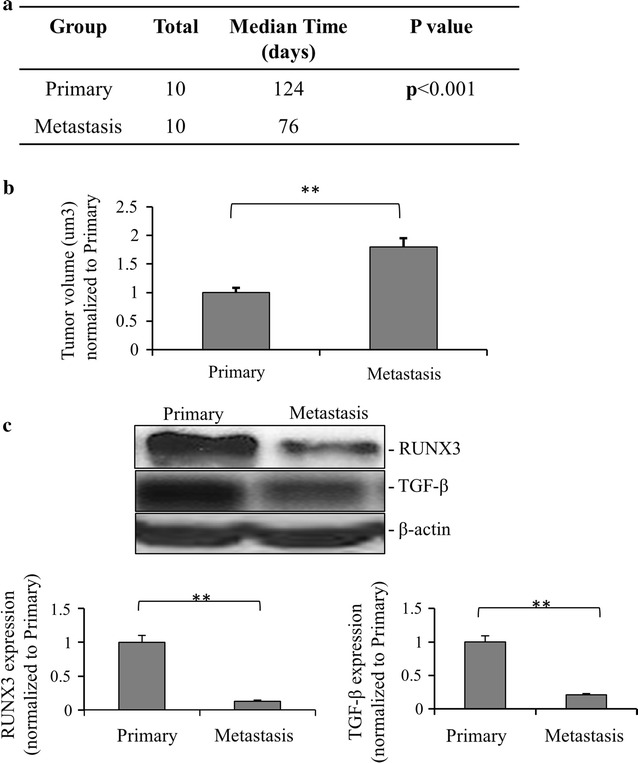
The downregulation of RUNX3 and TGF-β in murine renal tumor xenograft model in vivo. **a** The xenograft renal tumor metastasis and primary models were established after injection of 1 × 10^4^ corresponding cells in the left foreleg of mice, respectively. The survival days were calculated after each mouse was dead (n = 10, *p *< *0.01*). **b** Tumor volumes in xenograft models inoculated from renal metastatic tumor cells and primary tumor cells were measured 2 months post inoculation while all the mice were still alive but sick. Values are presented as mean ± SD from at least three independent experiments (**p < *0.01*). **c** RUNX3 and TGF-β expression in metastatic and primary renal cancer models were detected by Western blot. Relative expression values represent mean ± SD from three independent experiments (**p < *0.01*). Quantitation by densitometry is also shown

To investigate the roles of RUNX3 and TGF-β in metastatic renal cancer, we established two murine models, metastatic and primary renal cancer xenograft models by injection of 1 × 10^4^ corresponding renal cancer cells into the left forelegs of mice, respectively. About 3 months later, tumors had been formed and developed into late stages of the diseases. Mice were observed daily until they died of illness and the numbers of survival days were monitored. Figure 3a showed that compared with primary renal cancer xenograft model, the survival time of mice with metastatic renal cancer xenografts was significantly shortened, indicating that metastatic renal cancer has stronger lethality. 2 months post inoculation, animals with renal cancer xenografts formed clear tumors but no death observed. For calculating the sizes of the tumors, mice were sacrificed 2 months post inoculation. Figure 3b showed that the volumes of metastatic xenografts were significantly larger than the non-metastatic xenografts, indicating that the metastatic renal cancer had stronger proliferation and growth rate. Importantly, the expressions of RUNX3 and TGF-β were significantly lower in metastatic renal cancer model than those in non-metastatic renal cancer model. These results suggested that the RUNX3-TGF-β pathway played a role in the metastasis of renal cancer.

### Elevated CpG methylation of RUNX3 and TGF-β in murine metastatic renal cancer xenograft model

**Fig. 4:**
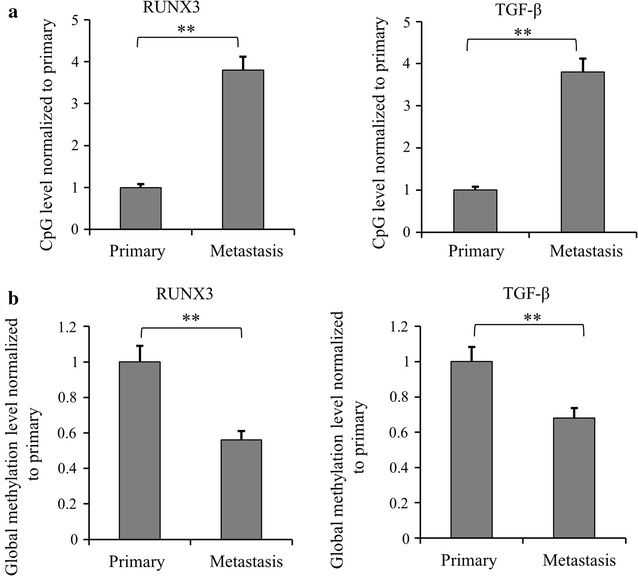
The methylation levels of RUNX3 and TGF-β in metastatic vs. primary renal cancer xenograft model. **a** CpG methylation levels of RUNX3 and TGF-β in metastatic xenograft model was detected by using qMSP compared with primary renal xenograft cancer model (**p < *0.01*). The data were mean ± SD of three independent experiments. **b** Global DNA methylation levels of RUNX3 and TGF-β in metastatic xenograft model were detected by using quantitative methylation real-time PCR compared with primary renal xenograft cancer model (**p < *0.01*). The data were mean ± SD of three independent experiments

Then we examined the methylation levels for RUNX3 and TGF-β in murine metastatic renal cancer xenograft model. The CpG methylations of RUNX3 and TGF-β were increased in the metastatic renal cancer model (Fig. 4a), while the global methylations of RUNX3 and TGF-β were decreased (Fig. 4b), consistent with the human cancer tissue results. As both RUNX3 and TGF-β play a tumor suppressor role in cancer development, their elevated CpG methylations inhibited their expression in metastatic cancer model thereby contributing to higher degree of malignancy.

### Inhibition of methylation increases the expression of RUNX3 and TGF-β and inhibits tumor malignancy

**Fig. 5:**
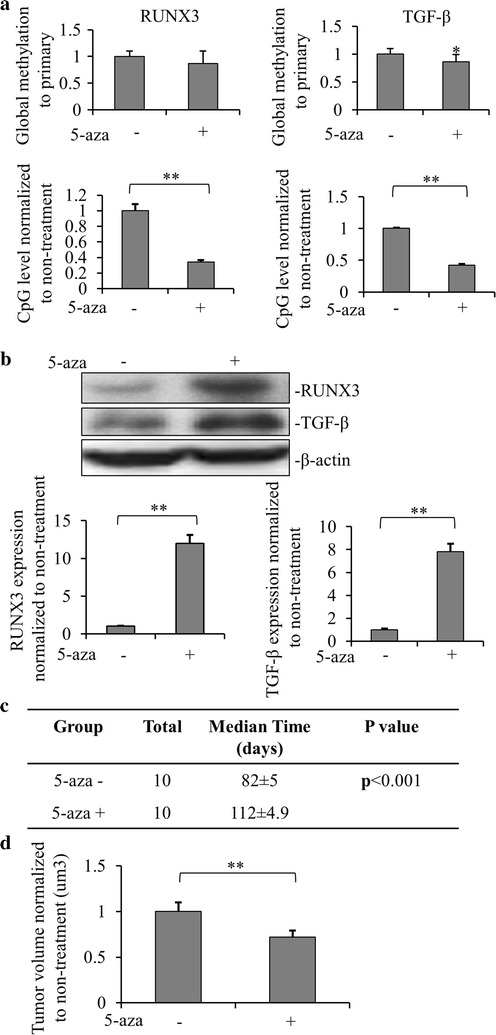
Demethylation suppressed the development of renal cancer by activating RUNX3 and TGF-β. **a** Global and CpG methylation levels of RUNX3 and TGF-β in metastatic xenograft cancer model inoculated with 5-aza-2′-deoxycytidine (10 mM) treated metastatic cancer cells were detected by using quantitative methylation real-time PCR and qMSP compared with non-treated metastatic xenograft models (*p < *0.05*, **p < *0.01*). The data were mean ± SD of three independent experiments. **b** RUNX3 and TGF-β expression with treatment of 5-aza-2′-deoxycytidine were detected in metastasis renal xenograft cancer model by Western blot. Relative expression values represent mean ± SD from three independent experiments (**p < *0.01*). Quantitation by densitometry is also shown. **c** The xenograft metastatic models with or without 5-aza-2′-deoxycytidine treatment was established after injection of 1 × 10^4^ different cells in the left foreleg, respectively. The survival periods were calculated after each mouse was dead (n = 10). **d** Tumor volumes were measured in xenografts from renal metastatic cancer cells with or without 5-aza-2′-deoxycytidine treatment 2 months post inoculation when mice were still alive but sick. Values are mean ± SD from at least three independent experiments (**p < *0.01*)

As our previous data showed that the high CpG methylation levels of RUNX3 and TGF-β played an important role in the metastasis ability of renal cancer, we next investigated whether decreased level of methylation by methylated transferase inhibitor methyltransferase (5-aza-2′-deoxycytidine) has an effect in the murine metastatic renal cancer xenograft model. 1 × 10^4^ metastatic renal cancer cells with 5-aza-2′-deoxycytidine treatment or untreated control were injected into the left foreleg of mice. Figure 5a showed that the CpG methylation levels of RUNX3 and TGF-β genes were decreased by 5-aza-2′-deoxycytidine treatment in metastatic renal cancer animal model. As expected, the expressions of RUNX3 and TGF-β were increased in 5-aza-2′-deoxycytidine treated metastatic renal cancer model by immunoblotting (Fig. 5b). Compared with the untreated group, the survival time of the mice inoculated with methylation inhibited metastatic renal cancer cells were significantly prolonged (Fig. 5c). At 2 months after inoculation, the sizes of the tumors were calculated. Figure 5d showed that the tumor size of the mice inoculated with 5-aza-2′-deoxycytidine treated metastatic renal cancer cells was significantly lower than that of the non-treated metastatic renal cancer cells. These results strongly indicated that inhibition of methylation was the key to inhibit the development of renal cell cancer through elevation of TGF-β/RUNX3 pathway. In addition, another DNA methylation inhibitor, SGI-1027, was selected to verify the effects of DNA methylation on suppressing tumor (Additional file 1: Figure S1), and the results confirmed that demethylation suppressed the development of renal cancer. Furthermore, we also found that the down-regulated TGF-beta/RUNX3 exhibited no significant effects on CpG methylation and global methylation (Additional file 1: Figure S2), which further suggested that the effect of methylation on the development of renal cancer was associated with TGF-beta/RUNX3 pathway inhibition.

## Discussion

In this study, we investigated the differentially expressed genes in metastatic versus primary renal cancer tissues. Most of the differentially expressed genes were downregulated, of which RUNX3 and TGF-β were top two downregulated genes in metastatic renal cancer with hypermethylation in CpG islands, while a hypomethylation was observed in the DNA levels of these two genes. Metastatic renal cancer xenograft model as well as primary renal cancer xenograft model was established by inoculation of the corresponding cancer cells to the forelegs of immunodeficient mice. Lower RUNX3 and TGF-β expressions, elevated CpG methylations as well as less global methylations in the DNA level were observed in the metastatic model. These results are consistent with the human tissue data. The metastatic cancer cells were more malignant, as demonstrated by the fact that bigger volume of tumors and shorter survival time of animals seen in the metastatic renal cancer xenograft model. Pretreatment of the cancer cells by the methylation inhibitor, 5-aza-2′-deoxycytidine before inoculation decreased the tumor size, extended the animal survival time with decreased methylation of CpG islands in the promoters of RUNX3 and TGF-β. This study strongly suggested that methylation played a critical role in regulating the metastasis of renal cancer, and is also associated with TGF-β/RUNX3 inhibition.

Methylation has been implicated in many cancer types. Methylation decreased the expression of bridging integrator-1 (Bin1) in esophageal squamous cell carcinoma, and Bin1 methylation could augment the malignant biological behaviors of esophageal squamous cell carcinoma [18]. Epigenetic silencing of Wnt antagonists, including secreted frizzled-related protein 2 (SFRP2) and dickkopf WNT signaling pathway inhibitor 2 (DKK2) was associated with gastric carcinogenesis, and concurrent hypermethylation of SFRP2 and DKK2 could be a potential marker for a prognosis of poor overall survival [19].

Three families of runt-related genes, RUNX1 (PEBP2 B/CBFA2/AML1), RUNX2 (PEBP2 A/CBFA1/AML3) and RUNX3 (PEBP2 C/CBFA3/AML2) exist in mammals. All three protein families play important roles in normal developmental processes as well as carcinogenesis [6, 20, 21]. RUNX3 is known to be a tumor suppressor exhibiting anti-tumor activity in several cancers. In our study, we found that RUNX3 expression was reduced in metastatic renal cancer tissues compared with primary renal cancer tissues, consistent with the previous studies. RUNX3 expression was reported to be lower in renal cell carcinoma tissue than in adjacent normal renal tissues, and RUNX3 targeted miR-6780a-5p/E-cadherin/EMT signaling axis to suppress migration and invasion of renal carcinoma cells [8]. Restoration of RUNX3 significantly decreased renal carcinoma cell migration and invasion capacity [9]. In another report, expression of RUNX3 was significantly decreased in 75 cases of clear cell renal cell carcinoma tissues, and RUNX3 inhibited the proliferative and metastatic abilities of clear cell renal cell carcinoma cells by regulating cyclins and TIMP1 [7]. As it has been strongly indicated that RUNX3 regulated the renal cancer cell metastasis in the previous studies, we studied the role of RUNX3 in renal cancer metastasis by comparison of metastatic and primary renal cancer.

The down-regulated expression of RUNX3 by hypermethylation occurs in its promoter region. RUNX3 is located on the short arm 1p36.1 of human chromosome 1, and its mRNA is mainly transcribed by P2 promoter, which is rich in CG content (64%) being a typical CpG island [22]. RUNX3 gene was reported to be inactivated by aberrant methylation in gastric, colorectal, bile duct, pancreatic and renal cancers [6, 22–25]. Our study is consistent with the previous results, as RUN3X downregulation by methylation in CpG islands was found in metastatic renal cancer tissues compared to primary renal cancer tissues.

The RUNX proteins are transcription factors targeting the TGF-β signaling pathway. RUNX3 translocates into the nucleus in response to TGF-β signal transduction, and may function in the nucleus as tumor suppressor and transcriptional regulator [11]. RUNX proteins have been shown to interact with downstream SMAD protein in mediating the growth-suppressive effects of TGF-β [26]. TGF-β was reported to induce epithelial-mesenchymal transition, migration and invasion in cancer cells [27, 28].

Consistently, our metastatic renal cancer xenograft model showed decreased expressions of both RUNX3 and TGF-β, which was caused by the methylation in their CpG islands. When the expressions of RUNX3 and TGF-β were restored by treatment with 5-aza-2′-deoxycytidine before inoculation into the in vivo model, the tumor size was smaller and the animal survival time was prolonged. These results indicated that the expressions of RUNX3 and TGF-β were regulated by methylation in CpG islands in their promoters. More importantly, it strongly indicated that the malignancy of metastatic renal cancer tissues could be weakened by introducing higher expressions of RUNX3 and TGF-β by inhibiting their promoter methylation. These findings provide new insights into the significant regulation mechanism of two cancer suppressor genes, RUNX3 and TGF-β in the metastasis of renal cancer, which also suggests an important role for methylation regulation in renal cancer metastasis. RUNX3 and TGF-β may also function as biomarkers for renal cancer metastasis prediction, and for selection of patient specific treatment plans. However, there are some limitations of the study: (1) methylation inhibition was achieved by global inhibition rather than locus-specific inhibition, hence the observed effect on TGF-β/RUNX3 expression might also be caused by other proteins down-regulated by global inhibition. More complex and direct experiments such as dCas9-mediated inhibition are warranted to address this question in the future. (2) TGF-β/RUNX3 knockdown or overexpression was not performed to directly analyze the effect on metastasis of renal cancer, which warrants further study using either genetically manipulated patient primary tissue cells or transgenic mouse models.

## Conclusions

This study has demonstrated that methylation regulates metastasis of renal cancer and is associated with TGF-β/RUNX3 pathway inhibition, with evidences from both patient renal cancer tissues and murine renal cancer xenograft models. It suggests the application of methylation suppression on tumor-related genes as a therapeutic strategy for renal cancer.

## Additional file

**Additional file 1: Table S1.** The location of patient with metastasizing cancer. **Figure S1.** Demethylation suppressed the development of renal cancer. **Figure S2.** There was no change of methylation levels in metastatic renal cancer tissue after RUNX3 and TGF-β knockdown.

## Abbreviations

TGF-β: transforming growth factor (TGF)-β
qMSP: quantitative methylation specific PCR
RUNX3: runt-related transcription factor 3
Bin1: bridging integrator-1
SFRP2: secreted frizzled-related protein 2
DKK2: dickkopf WNT signaling pathway inhibitor 2
