# Supplementary material for: DNA methylation affects metastasis of renal cancer and is associated with TGF-β/RUNX3 inhibition
Source: Cancer Cell Int. 2018 Apr 10;18:56. doi: 10.1186/s12935-018-0554-7 (PMC5894227; doi:10.1186/s12935-018-0554-7)
Supplement: Supplementary file 1 — Additional file 1: Table S1. The location of patient with metastasizing cancer. Figure S1. Demethylation suppressed the development of renal cancer. Figure S2. There was no change of methylation levels in metastatic renal cancer tissue after RUNX3 and TGF-β knockdown. [file 12935_2018_554_MOESM1_ESM.docx]

**Additional file**

**Table S1. The location of patient with metastasizing cancer (n=67)**

| Group | Kidney | Bone | Liver | Lung | lymph | Others |
| --- | --- | --- | --- | --- | --- | --- |
| Primary | 53 | 0 | 0 | 0 | 0 | 0 |
| Metastasis | 0 | 23 | 16 | 18 | 31 | 5 |

**Note: metastasizing cancer of some patients were in more than one places.**

**
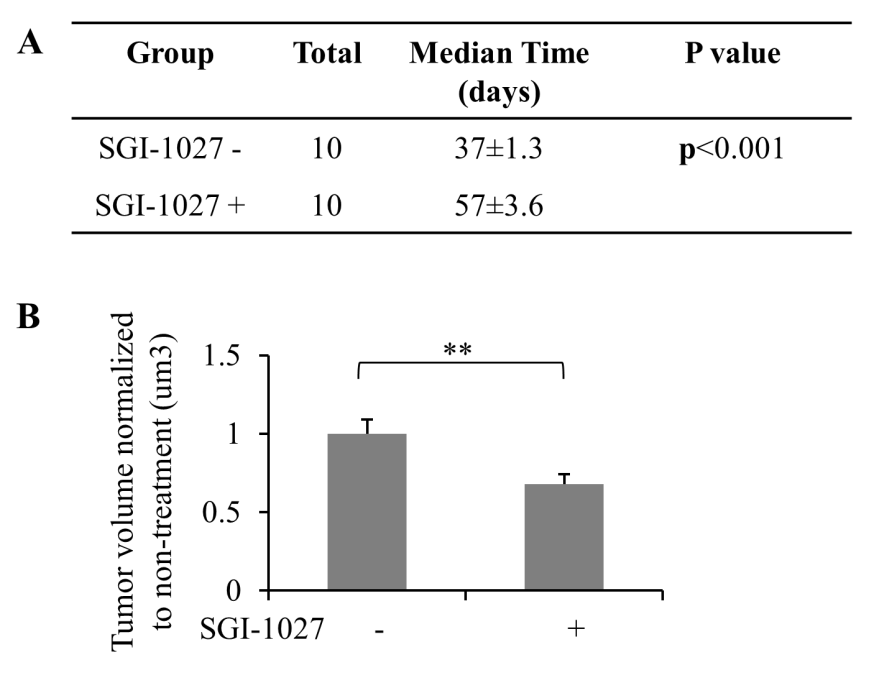
**

Figure S1. Demethylation suppressed the development of renal cancer. **A.** The xenograft metastatic models with or without SGI-1027 (10 μM) treatment were established after injection of 5×10^4^ different cells in the left foreleg, respectively. The survival periods were calculated after each mouse (n=10)*.* **B.** Tumor volumes were measured in xenografts from renal metastatic cancer cells with or without SGI-1027 treatment 1 month post inoculation when mice were still alive but sick. Values are mean ± SD from at least three independent experiments (***p<0.01*).


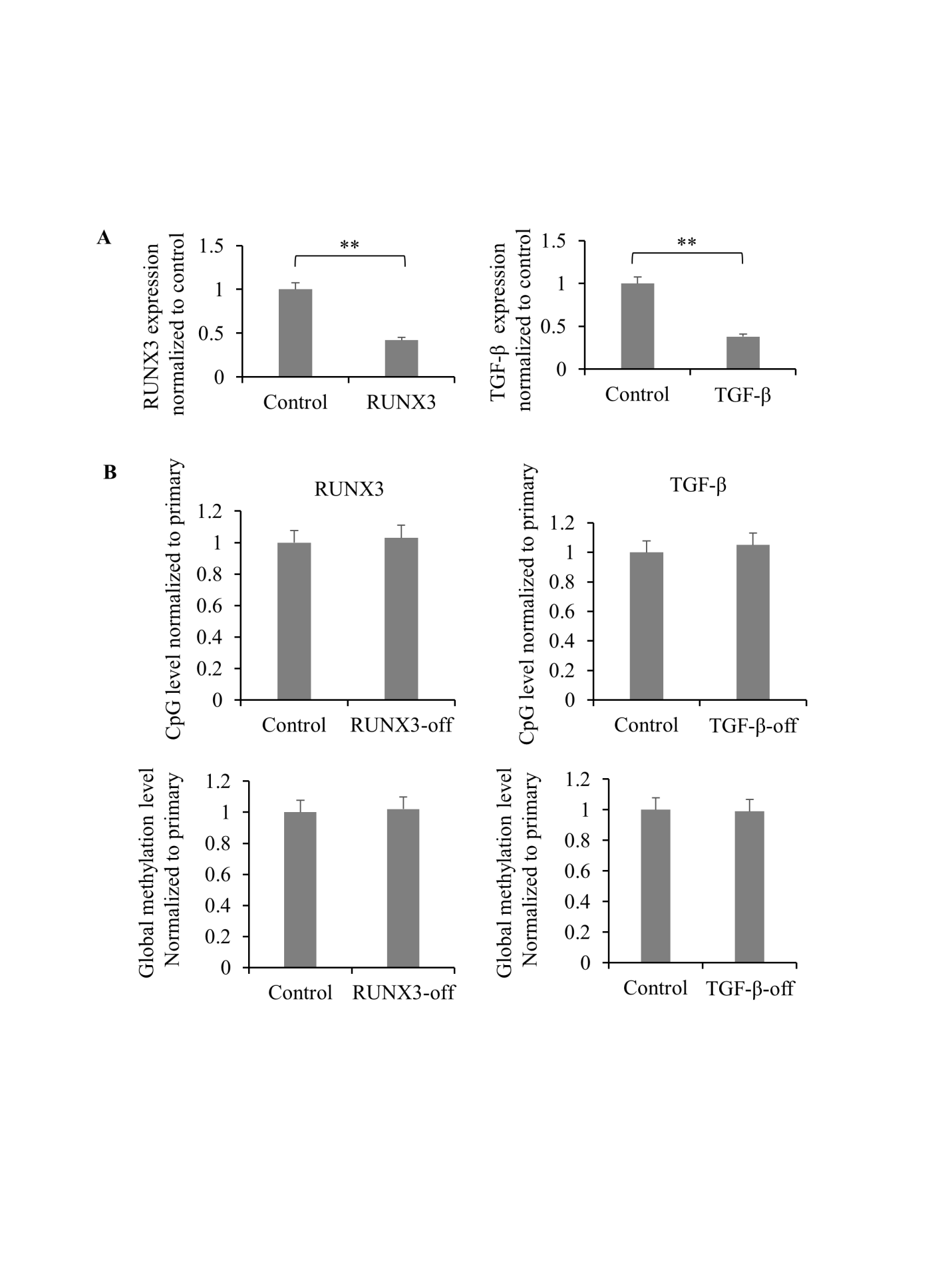


Figure S2. **No change of methylation levels in metastatic renal cancer tissue after RUNX3 and TGF-β knockdown**. **A.** The protein levels of RUNX3 and TGF-β were detected by RT-PCR after RUNX3 and TGF-β knockdown. The data were mean ± SD from three independent experiments (***p<0.01*). **B.** CpG methylation levels of RUNX3 and TGF-β in metastatic cancer tissues were detected by qMSP after RUNX3 and TGF-β knockdown. The data were mean ± SD from three independent experiments. Then global DNA methylation levels of RUNX3 and TGF-β in metastatic renal cancer tissues were detected using quantitative methylation real-time PCR after RUNX3 and TGF-β knockdown. The data were mean ± SD from three independent experiments.
